# CrowdAttention: An Attention Based Framework to Classify Crowdsourced Data in Medical Scenarios

**DOI:** 10.3390/s25206435

**Published:** 2025-10-17

**Authors:** Julian Gil-Gonzalez, David Cárdenas-Peña, Álvaro A. Orozco, German Castellanos-Dominguez, Andrés Marino Álvarez-Meza

**Affiliations:** 1 Automatics Research Group, Universidad Tencológica de Pereira, Pereira 660003, Colombia; dcardenasp@utp.edu.co (D.C.-P.); aaog@utp.edu.co (Á.A.O.); 2 Signal Processing and Recognition Group, Universidad Nacional de Colombia, Sede Manizales, Manizales 170003, Colombia; cgcastellanosd@unal.edu.co (G.C.-D.); amalvarezme@unal.edu.co (A.M.Á.-M.)

**Keywords:** learning from crowds, attention mechanisms, deep learning, classification

## Abstract

Supervised learning models in healthcare and other domains heavily depend on high-quality, labeled data. However, acquiring expert-verified labels (i.e., the gold standard) is often impractical due to cost, time, and subjectivity. Crowdsourcing offers a scalable alternative by collecting labels from multiple non-expert annotators; however, it introduces label noise due to the heterogeneity of annotators. In this work, we propose *CrowdAttention*, a novel end-to-end deep learning framework that jointly models classification and annotator reliability using a cross-attention mechanism. The architecture consists of two coupled networks: a classification network that estimates the latent true label, and a crowd network that assigns instance-dependent reliability scores to each annotator’s label based on its alignment with the model’s current prediction. We demonstrate the effectiveness of our approach on both synthetic and real-world datasets, showing improved accuracy and robustness compared to state-of-the-art multi-annotator learning methods.

## 1. Introduction

In recent years, Deep Learning (DL) has transformed healthcare applications, including disease prediction, drug discovery, and medical image analysis [1]. These applications typically rely on supervised learning algorithms that can learn complex representations when trained on extensive labeled data collections. Consequently, the performance of DL models is closely linked to the quality of the training labels, which are often assumed to represent the absolute ground truth [2]. However, in many scenarios, obtaining such ground truth is hardly feasible because the labeling process is costly and time-consuming; moreover, in some cases, the label corresponds to a subjective assessment [3]. The above challenge is particularly acute in the medical domain, where specialists are scarce and their time is highly valuable [4]. To address this bottleneck and improve scalability, many initiatives rely on data labeled by multiple annotators with varying expertise levels via crowdsourcing. The main idea is to distribute the annotation effort among different labelers (e.g., physicians), reducing the burden on experts [5]. Nevertheless, differences in expertise, annotation criteria, and data interpretation can introduce label variability and noise, which can degrade model performance if not correctly handled [6,7]. As a result, in the case of crowdsourcing labels, each instance in the training set is assigned to a set of multiple annotators corresponding to a noisy version of the hidden gold standard [8]. Accordingly, it is necessary to develop methods for dealing with labels from multiple annotators, known in the literature as *Learning from Crowds* (LFC) [9]. LFC can be divided into two perspectives: label aggregation and training DL under supervision from all labelers [8].

In particular, label aggregation configures a two-stage training procedure: the first step comprises estimating a single label, assumed to approximate the ground truth, which is then used to train a standard DL algorithm [10]. The most naive method is the so-called majority voting (MV); however, MV assumes annotators with homogeneous performance, which is not feasible due to the presence of annotators with different expertise levels [11]. Thus, elaborated approaches have been proposed to compute the annotators’ performance while producing the ground truth estimation. For example, the early work in [12] treats the ground truth as a latent variable. It uses an Expectation-Maximization (EM) algorithm to jointly estimate the annotators’ performance and the hidden ground truth. Similarly, in [13], the authors propose a weighted MV aiming to code the heterogeneous performances. Despite their simplicity, most label aggregation methods ignore the input features, missing crucial details [14].

In contrast, a more integrated alternative is to adopt end-to-end architectures to train DL models directly from the multi-annotator labels. The basic idea is to modify typical DL models, including *Crowd Layers* [15], to jointly estimate the supervised learning algorithm and the annotators’ behavior. Several works have shown that this second approach improves performance, as input features provide valuable information to infer the true labels [16]. The success of end-to-end models relies on the proper codification of the annotators’ performance, which, for classification settings, is usually measured in terms of accuracy [17], sensitivity, specificity [11], or the confusion matrix [2,18]. Nevertheless, a restriction commonly found in the above approaches is that they assume that the performance is homogeneous, which does not hold in real scenarios, because the labels are provided based not only on the labelers’ expertise but also on the features observed from raw data [11]. Hence, the model proposed in [19] is the first attempt to relax this assumption. Here, the annotations provided by each source are modeled by a Bernoulli distribution, where the probability parameter is a function of the input space, which is related to the performance of that annotator. This seminal work has inspired more elaborate models that employ multi-output (MOL) learning concepts to model the annotators’ parameters as a function of the input features [20]. Unlike standard multi-output learning problems, where each output corresponds to a task, each annotator is modeled as a separate function whose parameters depend on the input, capturing heterogeneous behavior [4].

While recent models have extended the standard multi-output framework to jointly estimate both the ground truth and annotator reliability as functions of the input, and even capture correlations among annotators, they face important limitations in terms of scalability. These architectures use dedicated outputs for each annotator, which allows modeling of inter-labeler dependencies but results in several parameters and computations that grow linearly (or worse) with the number of annotators. This can become prohibitive in large-scale crowdsourcing settings, where the pool of annotators is numerous [5,21].

To address the scalability and robustness challenges described above, we introduce *CrowdAttention*, a novel end-to-end deep learning framework designed to learn from noisy labels provided by multiple annotators. The architecture comprises two main components: a classification network that produces the final prediction, and a crowd network that estimates the latent ground truth through a cross-attention mechanism over the annotators’ responses. The classification network maps the input features to a distribution over class labels. In parallel, this predicted distribution is used as the *query* in a cross-attention module, while the one-hot encoded responses from multiple annotators serve as *keys* and *values*. Crucially, the attention mechanism assigns instance-dependent weights that explicitly represent the reliability of each annotator, thereby producing a pseudo-label that reflects a reliability-weighted consensus rather than a simple majority vote. The model is trained by minimizing a cross-entropy loss between the classification network’s prediction and the pseudo-label produced by the annotators’ network, which allows a joint optimization of both components. This training strategy allows the model to benefit from various labelers while avoiding the need for manually aggregated labels or architectural replication for each annotator.

In summary, our main contributions are as follows:

- We propose *CrowdAttention*, a scalable and parameter-efficient framework that models the reliability of multiple annotators as a function of the input space through a cross-attention mechanism.
- In contrast to previous methods [4,20,22], which rely on dedicated output branches for each annotator, our approach employs a unified attention-based design that substantially reduces the number of trainable parameters while enabling end-to-end training directly on datasets annotated by multiple annotators.
- We conduct comprehensive evaluations on synthetic and real-world datasets, demonstrating that *CrowdAttention* achieves superior classification performance and robustness under noisy and heterogeneous supervision, all while preserving architectural simplicity and a significantly lower parameter footprint.

The agenda is as follows: Section 2 presents the related work and paper contributions. Section 3 describes our proposal details. Section 4 and Section 5 present the results and discussion. Finally, Section 6 shows the concluding remarks.

## 2. Related Work

Typical supervised learning algorithms operate under the assumption that each training instance is associated with a single, reliable ground-truth label. This allows models to learn the relationship between inputs and outputs [11]. However, in many real-world applications, such as medical diagnosis or speech assessment, labels are often provided by multiple annotators. Their judgments can diverge due to differences in expertise, bias, or the ambiguity of the task [23]. This multi-annotator scenario results in collections of labels that are frequently noisy and, in many cases, incompatible with traditional supervised approaches. Rather than relying on a single source of truth, the learning procedure in these contexts requires explicitly modeling the annotators’ behavior to extract meaningful supervision from heterogeneous annotations.

The field of *learning from crowds* has emerged to address the supervision problem when data are annotated by multiple, and potentially unreliable, contributors. Applications cover a wide range from medical image analysis [24] and quality speech assessment [25] to natural language processing [26], where multiple annotators provide inconsistent labels. In this field, we identify two main approaches. The first is termed *label aggregation*, which comprises the estimation of a soft label for each training sample, which is then used to train a learning algorithm. Conversely, the second direction jointly estimates the latent ground truth and the parameters of a machine learning algorithm. This family of methods utilizes the interaction between annotator reliability and predictive performance, allowing the classifier and the label inference process to inform each other cohesively [14].

### 2.1. Label Aggregation

The simplest method to combine labels from multiple annotators is called *majority voting* (MV) [27]. This approach selects the most frequent label as a soft estimation of the hidden ground truth. However, MV assumes that all annotators perform at a similar level, which often does not reflect reality. In real-world applications, factors such as expertise, bias, and reliability can vary significantly among labelers. To address this, several variants have been proposed. For example, the Iterative Weighted Majority Voting (IWMV) [28] starts by applying MV to obtain initial aggregated labels. These labels are then used to estimate the reliability of each annotator. The reliability estimates serve as weights in subsequent rounds of aggregation, allowing the method to iteratively update both the annotator weights and the integrated labels until convergence is achieved. Some extensions utilize the confidence of the majority class. For instance, ref. [29] presents four soft-MV schemes that integrate certainty information to generate more informative consensus labels. Additionally, ref. [30] uses differential evolution to assess the quality of annotators based on multiple sets of noisy labels. Additionally, the Label Augmented and Weighted Majority Voting (LAWMV) [31] method leverages K-nearest neighbors to enrich each instance’s label set and applies weighted majority voting based on distance and label similarity.

Beyond heuristic refinements of MV, probabilistic aggregation models have been developed. The seminal work proposed in [12] jointly estimates the latent ground truth and each annotator’s confusion matrix using an expectation–maximization algorithm, allowing the model to weight annotations according to estimated reliability. Subsequent extensions, such as the Generative model of Labels, Abilities, and Difficulties (GLAD) [32], further incorporate item difficulty and annotator expertise, capturing the intuition that some annotators are more accurate on specific tasks and that certain items are intrinsically harder to label. Similarly, in [33], the authors generate multiple clusters based on the information from multiple annotators; then, each cluster is assigned to a specific class to solve a multi-class classification problem.

### 2.2. End-to-End Algorithms

Despite their simplicity, most label aggregation methods ignore the input features, missing crucial details to puzzle out the ground truth [14]. Alike, an alternative is to adopt end-to-end architectures to learn DL models directly from the multi-labeler data. The basic idea is to modify the last layer in DL models in order to include an additional *Crowd Layer* [15], which codes the annotators’ performance. Thereby, the classifier and the *Crowd Layer* can be estimated simultaneously via backpropagation [15]. Following this idea, several works such as [14,34,35] propose a probabilistic model, where the main idea is to model each labeler via a confusion matrix $M\in R^{K\times K}$, being *K* the number of classes. Such matrices are fed by the output of a basic model, which represents an estimation of the hidden ground truth. Therefore, the labels of each annotator can be seen as a modification of the ground truth with varying levels of reliability and bias [14].

However, these methods typically assume that transition matrices are annotator- and instance-independent, which is unrealistic in practical scenarios. To relax this assumption, ref. [18] estimates annotator- and instance-dependent transition matrices using deep neural networks combined with knowledge transfer strategies. In this framework, knowledge about the mixed noise patterns of all annotators is first extracted and then transferred to individual workers. At the same time, additional refinement is achieved by transferring information from neighboring annotators to improve the estimation of each annotator’s transition matrix. On the other hand, recent works such as [20,22], categorized as Chained Neural Networks (Chained-NN), employ a framework based on the concept of *Chained Gaussian Processes* [36] to model annotator performance as a function of the input features. The central idea is to design a neural network with K+R outputs, where *K* outputs correspond to the underlying classification task and each of the remaining *R* outputs (one per annotator) captures the annotator-specific reliability. In this way, the model simultaneously learns the predictive function and the input-dependent reliability of each labeler, thereby relaxing the assumption of homogeneous annotator performance. Despite their improved flexibility, the previous approaches model the annotators’ performance using a parametric network. For example, transition matrix-based algorithms require R×K×K parameters for *R* annotators, which can lead to learning inefficiencies for a large number of annotators or classes [5].

### 2.3. Main Contribution

According to the above, we identify that most existing approaches share a standard limitation: they model annotator performance using parametric forms that either assume homogeneity or require large parameter spaces to capture heterogeneity. This motivates the need for alternative formulations that can efficiently represent annotator variability, adapt to input-dependent reliability, and scale to realistic multi-annotator scenarios.

We propose CrowdAttention, an end-to-end framework that integrates attention mechanisms to model annotators’ reliability as a function of the input features. The architecture comprises two main components: a classification network that produces the final prediction, and a crowd network that estimates the latent ground truth through a cross-attention mechanism over the annotators’ responses. The classification network maps the input features to a distribution over class labels. In parallel, this predicted distribution is used as the *query* in a cross-attention module, while the one-hot encoded responses from multiple annotators serve as *keys* and *values*. The attention mechanism dynamically weighs each annotator’s contribution, producing a pseudo-label that reflects the annotators’ consensus weighted by instance-specific relevance.

In contrast to confusion–matrix–based approaches [12,14,32], which require R×K×K parameters to capture annotator variability, CrowdAttention avoids explicit parameterization of each annotator’s noise process. Instead, annotator reliability is modeled in a non-parametric manner through a dot-product attention mechanism learned jointly with the classification network. This results in a compact representation that flexibly adapts across annotators and instances without introducing large parameter overheads. Furthermore, unlike *Chained* approaches [20,22], which also account for annotator reliability as a function of inputs but remain computationally demanding, CrowdAttention embeds a lightweight attention mechanism within modern deep architectures, ensuring both scalability to large crowdsourcing datasets and robustness to heterogeneous supervision.

Furthermore, our *CrowdAttention* shares similarities with the work in [5] in the sense that both approaches aim to improve training efficiency by eliminating the need for multiple annotator-specific transition matrices. Instead of requiring R×K×K parameters, they adopt a more compact representation that aggregates annotator information within an end-to-end framework. However, unlike UnionNet, which concatenates all annotators’ labels into a single union and relies on a parametric transition matrix, *CrowdAttention* employs a non-parametric dot-product attention mechanism that models the labelers as a function of the input space. Table 1 summarizes the key insights from our proposal alongside state-of-the-art methods.

## 3. Background

### 3.1. Problem Formulation

In standard deep learning settings, a *K*-class classification task consists of estimating a function $f_{\theta }:X\to Y$, where X denotes the input space, and $Y={\left\{0,1\right\}}^{K}$ is the space of one-hot encoded class labels. The function is parameterized by θ and trained using a dataset $D={\left\{\left(x_{n},y_{n}\right)\right\}}_{n=1}^{N}$, where $x_{n}\in X$ and $y_{n}\in Y$. In crowdsourced classification, the true labels $y_{n}$ are not available. Instead, each input $x_{n}$ is annotated by a subset of labelers $W_{n}\subseteq \left\{1,\ldots ,W\right\}$, each providing a possibly noisy label. Let $o_{n}^{\left(w\right)}\in Y$ denote the one-hot encoded label assigned by annotator $w\in W_{n}$ to instance *n*. We assume that every instance is labeled by at least one annotator, i.e., $|W_{n}|\geq 1$ for all *n*. Consequently, the observed dataset can be formalized as follows:

(1) $$D_{R}={\left\{\left(x_{n},{\left\{o_{n}^{\left(w\right)}\right\}}_{w\in W_{n}}\right)\right\}}_{n=1}^{N}.$$

where $o_{n}^{\left(w\right)}\in {\left\{0,1\right\}}^{K}$. Our objective is to estimate the latent true labels $Y=[y_{1}^{⊤},\ldots ,y_{N}^{⊤}]$ and to learn the reliability of each annotator as a function of the input features.

### 3.2. CrowdAttention Framework

To address the challenges posed by noisy and heterogeneous annotations in crowdsourced data, we introduce *CrowdAttention*, a flexible and scalable framework for learning from multiple imperfect labelers. CrowdAttention relies on two coupled neural networks. The first is a *classification network* that maps the input instance $x_{n}$ to a class probability distribution ${\hat{y}}_{n}\in {\left[0,1\right]}^{K}$. The second is a *crowd network* that processes the annotator-provided labels $\left\{o_{n}^{\left(w\right)}\right\}$ to build a soft approximation of the hidden gold standard ${\tilde{y}}_{n}\in {\left[0,1\right]}^{K}$.

**Figure 1:**
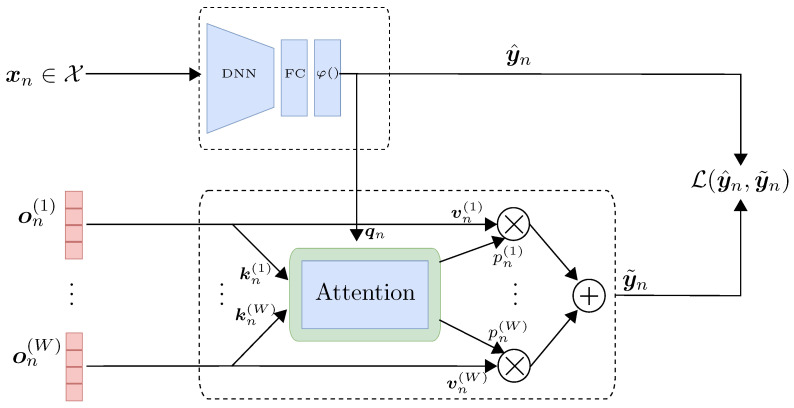
CrowdAttention framework. It comprises a classification network for latent expert predictions and a crowd network that estimates annotator reliabilities for informed label aggregation. For each instance $x_{n}$, a deep neural network outputs a prediction ${\hat{y}}_{n}$, serving as a soft approximation of the hidden ground truth. The one-hot encoded annotations $o_{n}^{\left(w\right)}\in {\left\{0,1\right\}}^{K}$ from multiple annotators are mapped into keys $k_{n}^{\left(w\right)}$ and values $v_{n}^{\left(w\right)}$. The query vector $q_{n}={\hat{y}}_{n}$ guides the attention mechanism to assign weights $p_{n}^{\left(w\right)}$, which represent the reliability of the *w*-th annotator and are used to generate the aggregated label ${\tilde{y}}_{n}$. The model is trained by minimizing the loss $L({\hat{y}}_{n},{\tilde{y}}_{n})$.

As shown in Figure 1, which provides an overview of the model components, rather than defining an independent network to estimate annotator-specific transition matrices [17] or reliabilities [4,22], we propose a shared model based on the *attention mechanism*. This approach enables the estimation of annotator-specific reliabilities as instance-dependent variables while maintaining a compact architecture.

Then, inspired by prior works that model annotator quality in terms of reliability [17,22], we define a continuous variable $p_{n}^{\left(w\right)}\in \left[0,1\right]$ for each instance $x_{n}$ and annotator $w\in W_{n}$. This variable reflects the degree of trust assigned to the label provided by annotator *w*, conditioned on both the input and the observed annotations. In this section, we abstract the computation of $p_{n}^{\left(w\right)}$, which is derived from an attention mechanism that will be described in detail. Formally, given the set of noisy labels ${\left\{o_{n}^{\left(w\right)}\right\}}_{w\in W_{n}}$, we define the pseudo-label ${\tilde{y}}_{n}\in {\left[0,1\right]}^{K}$ as follows:

(2) $${\tilde{y}}_{n}=\frac{1}{\sum_{w\in W_{n}}p_{n}^{\left(w\right)}}\sum_{w\in W_{n}}p_{n}^{\left(w\right)}o_{n}^{\left(w\right)},$$

where the normalization ensures that ${\tilde{y}}_{n}$ forms a valid probability distribution. Our *CrowdAttention* allows for emphasizing annotations from more reliable annotators, without requiring access to ground-truth labels or the estimation of per-annotator parameters. The following subsection describes how these reliability scores are inferred using an attention-based module.

#### 3.2.1. Classification Network

The classification network in *CrowdAttention* maps each input instance $x_{n}$ to a predicted class distribution ${\hat{y}}_{n}$, which serves two purposes: it approximates the latent expert label and it is reused as the query vector in the attention-based crowd module. Formally, the network is built as the composition of two functions:

(3) $${\hat{y}}_{n}=f_{\theta }\left(x_{n}\right)=\varphi \left(g_{\theta_{2}}\left(h_{\theta_{1}}\left(x_{n}\right)\right)\right),$$

where $f_{\theta }$ denotes the complete model parameterized by θ. Additionally, $h_{\theta_{1}}:X\to R^{M}$ is a function that maps the inputs to a latent space $R^{M}$. Such a function represents the encoder, which can be instantiated as a multilayer perceptron, a convolutional network, or a transformer-based architecture, depending on the modality of the input. Conversely, $g_{\theta_{2}}:R^{M}\to R^{K}$ transforms the encoded features into class logits. These logits are then passed through the softmax activation $\varphi :R^{K}\to {\left[0,1\right]}^{K}$ to obtain the final predicted distribution.

At inference time, the classification network acts independently to produce the final prediction ${\hat{y}}_{n}$, which serves as the model’s estimation of the expert (latent) label distribution, without relying on annotator inputs [23].

#### 3.2.2. Crowd Network

The *crowd network* in *CrowdAttention* is responsible for computing instance-dependent reliability weights for the set of labels provided by multiple annotators. These reliability parameters, $p_{n}^{\left(w\right)}$, explicitly represent the confidence or trustworthiness assigned to annotator *w* for instance $x_{n}$, and are then used to compute the pseudo-label ${\tilde{y}}_{n}$ as in Equation (2). To estimate $p_{n}^{\left(w\right)}$, we adopt a cross-attention mechanism module expressed in the standard *query–key–value* (QKV) formalism. The *query* $q_{n}={\hat{y}}_{n}$ encodes the model’s current estimation regarding the gold standard. Additionally, the one-hot label from *w*-th annotator $o_{n}^{\left(w\right)}$ is assumed to be the *w*-th *key*, $k_{n}^{\left(w\right)}$.

By assessing the correlation between the ground-truth estimation (*query*) and the labels from multiple annotators (*keys*), the attention mechanism directly yields the reliability weights, i.e., $p_{n}^{\left(w\right)}=\xi \left(q_{n},k_{n}^{\left(w\right)}\right)$. In the literature, several approaches have been proposed as score functions [37]; however, in our setting, we instantiate ξ as a simple dot product:

(4) $$p_{n}^{\left(w\right)}=q_{n}^{\;⊤}k_{n}^{\left(w\right)}={\hat{y}}_{n}{\left(x_{n};f_{\theta }\right)}^{\;⊤}k_{n}^{\left(w\right)},$$

where the notation ${\hat{y}}_{n}\left(x_{n};\theta \right)$ highlights the dependence of the predicted class distribution concerning the classification network parameters in θ. The latter avoids additional parameters and computational overhead, which is critical in large-scale crowdsourcing settings. Additionally, unlike standard attention modules, we do not apply a softmax to the attention scores because the *keys* are one-hot vectors and the *query* lies in the probability simplex; hence, $p_{n}^{\left(w\right)}\in \left[0,1\right]$ is a normalized estimation of the annotators’ reliabilities.

Next, given the reliability parameters ${\left\{p_{n}^{\left(w\right)}\right\}}_{w=1}^{W}$, we use such weights to compute a weighted combination of the *values*, resulting in the final output. Here, we use the same one-hot vectors $o_{n}^{\left(w\right)}\in {\left\{0,1\right\}}^{K}$ as the *values*, resulting in the final output as defined in Equation (2).

In CrowdAttention, the cross-attention module operates with a single query and *W* annotators, each represented as a one-hot vector of dimension *K*. Hence, the computation of annotator scores requires O(WK) operations, and the weighted aggregation into the pseudo-label ${\tilde{y}}_{n}$ adds another O(WK). Hence, the overall complexity per instance is O(WK), which is substantially more efficient than methods based on annotator-specific transition matrices, whose cost grows quadratically with *K*.

#### 3.2.3. Loss Function

The *CrowdAttention* framework is trained end-to-end by minimizing a cross-entropy loss between the predicted class $\hat{y}$ distribution and the pseudo-label computed from the crowd network $\tilde{y}$. The total loss over the dataset $D_{R}$ is computed as follows:

(5) $$L\left(\theta \right)=-\frac{1}{N}\sum_{n=1}^{N}\sum_{k=1}^{K}{\tilde{y}}_{n,k}\log\left({\hat{y}}_{n,k}\right).$$

This training objective allows the model to update both the classification network and the attention-based crowd network jointly. By relying on soft labels ${\tilde{y}}_{n}$, the model leverages the consensus of multiple annotators while being robust to individual noise. Importantly, no ground-truth labels are required; learning proceeds directly from the noisy labels.

## 4. Experimental  Set-Up

### 4.1. Tested Datasets

In order to test our *CrowdAttention*, we use two kinds of datasets. First, we employ a fully synthetic example to show the capabilities of our approach to estimate the labelers’ performance, resulting in improved predictive accuracy. We define a three-class classification task. True labels are assigned based on the index of the maximum among the functions $\sin(2\pi x_{n})$, $-\sin(2\pi x_{n})$, and $0.5-\sin(2\pi \left(x_{n}+0.25\right))$. We generate 300 samples for training and 200 for testing, uniformly distributed over the interval [0,1]. Synthetic annotators are simulated following the procedure in [4], which generates correlated annotators whose reliabilities depend on the input features.

Second, to evaluate real-world applicability, we assess our model on two datasets sourced from clinical applications. The first dataset configures a classification task focused on detecting voice pathologies. Specifically, we use a subset of 218 samples of the Massachusetts Eye and Ear Infirmary Disordered Voice Database [38], which contains audio recordings from healthy individuals and patients with vocal disorders. Each recording is represented using Mel-frequency cepstral coefficients (MFCCs) as input features. Up to four medical experts rated the recordings using the GRBAS protocol, which evaluates five perceptual dimensions: Grade (G), Roughness (R), Breathiness (B), Asthenia (A), and Strain (S). Each dimension is scored on a scale from 0 (normal) to 3 (severe), thus defining five classification problems with K=4. However, following the analysis of annotator quality in [39], we exclude the Asthenia and Strain dimensions due to their poor reliability. Consequently, we retain only the G, R, and B dimensions, resulting in three multiclass classification problems with K=4. Finally, these problems are converted into binary classification tasks to align with the ground-truth labels available for evaluation.

For the second real-world experiment, we use a histopathology dataset derived from the TCGA Breast Cancer cohort [40]. The dataset includes 161 regions of interest (ROIs) extracted from 151 Whole Slide Images (WSIs). Two experienced pathologists provided the expert annotations, and the crowdsourced labels came from 20 medical students without pathology training. Experts and students were required to delimit regions such as tumors, stroma, and immune infiltrates [41]. Since WSIs are extremely large and cannot be directly processed by standard deep learning models, each WSI and its corresponding crowdsourced masks were divided into patches of size 224×224, following the preprocessing used in [2,41]. These patches define the input instances of our study. Although this dataset was initially designed for semantic segmentation, we follow [2,41] in redefining it as a multiclass classification problem with three classes: tumor, stroma, and immune infiltrates. The dataset is partitioned into a training set of 75,243 patches (with 108,495 crowdsourced annotations from medical students) and a test set of 4364 patches annotated by senior pathologists, which are considered the ground truth for supervised evaluation.

To further evaluate the generalization of our approach beyond the biomedical domain, we consider two additional benchmarks with real crowdsourced annotations. The first is a subset of the LabelMe dataset [42], following the protocol of [43], which contains 2688 natural images. A subset of 10,000 images was annotated on Amazon Mechanical Turk by 2.5 workers per image on average, across eight scene categories, and used for training, while the remaining 1688 images form the test set. The second is the Music Genre Database (MGC), which comprises 1000 audio clips of 30 s each, evenly distributed across ten genres. A subset of 700 clips was annotated on Amazon Mechanical Turk by multiple workers, and each clip is represented in a 124-dimensional feature space [17]. These datasets complement the medical tasks by introducing visual and audio modalities with different levels of annotator subjectivity, thus providing a broader test of robustness for multi-annotator learning.

### 4.2. Training Details and Method Comparison

As previously described, our proposed *CrowdAttention* framework consists of two coupled components: a classification network and a crowd network. The classification network can be instantiated with any deep learning architecture, depending on the nature of the input data. For the fully synthetic and voice datasets, we use a fully connected neural network with two hidden layers comprising 512 and 256 units, respectively, followed by ReLU activations and a softmax output layer. We employ a VGG-16 model pre-trained on ImageNet as a feature extractor for the histopathology dataset. We apply a global average pooling layer after the last convolutional block (size 7×7), which produces a 512-dimensional feature vector per patch. This vector feeds a fully connected classifier with a hidden layer of size 256 and an output layer adapted to the number of classes. All networks are trained using the Adam optimizer with a learning rate of 0.005 and a batch size of 1000 for the histopathology dataset; for the others, batch gradient descent (i.e., using all training samples per iteration) is used.

The overall accuracy (Acc) and the $F_{1}$ score carry out the quality assessment. Furthermore, a cross-validation scheme for the voice dataset is employed with 10 repetitions where 70% of the samples are utilized for training and the remaining for testing. The training and testing sets are clearly defined for the fully-synthetic and histopathology datasets. Table 2 displays the employed methods of the state-of-the-art for comparison purposes. The Python codes for our approach are available in https://github.com/juliangilg/CrowdAttention (accessed on 8 October 2025).

To provide a fair and interpretable comparison, we restricted the set of baseline methods, as shown in Table 2, to those that are both representative of the main methodological families and feasible to reproduce under our experimental setup. Specifically, we included majority-voting and EM-based aggregation methods (DNN-MV and DNN-DS), crowd-layer approaches (DLFC), chained architectures that model annotator reliability as a function of the input (CDL-CCE and CDL-GCE), and a recent scalable alternative based on concatenation (UnionNet). While other methods discussed in our Related Work (see Section 2) contribute important perspectives to the field, their functionality is conceptually similar to that of the included baselines, or their implementations were not publicly available, making a direct experimental comparison infeasible. This selection ensures that the reported results reflect the most relevant and reproducible baselines across the main directions of learning from crowds.

## 5. Results and Discussion

### 5.1. Fully-Synthetic Datasets Results

We first conducted a controlled experiment to evaluate the capabilities of our *CrowdAttention* in solving classification tasks while simultaneously estimating annotator performance as a function of the input features. For this purpose, we used the *fully synthetic* dataset described in Section 4.1. We simulated annotations from five labelers with varying levels of expertise, resulting in average accuracies (%) of [84.66,59.66,66.33,55.66,27.66].

After 10 iterations with different initializations, our method achieves an $F_{1}$-score of 97.70±0.43. This demonstrates the competitiveness of our approach, considering that the theoretical upper bound, represented by DNN-GOLD—a deep neural network trained directly on the true (gold-standard) labels—achieves 99.00±0.00. Remarkably, unlike DNN-GOLD, our method does not have access to the gold-standard labels, relying solely on the noisy annotations provided by multiple annotators with unknown levels of expertise.

**Figure 2:**
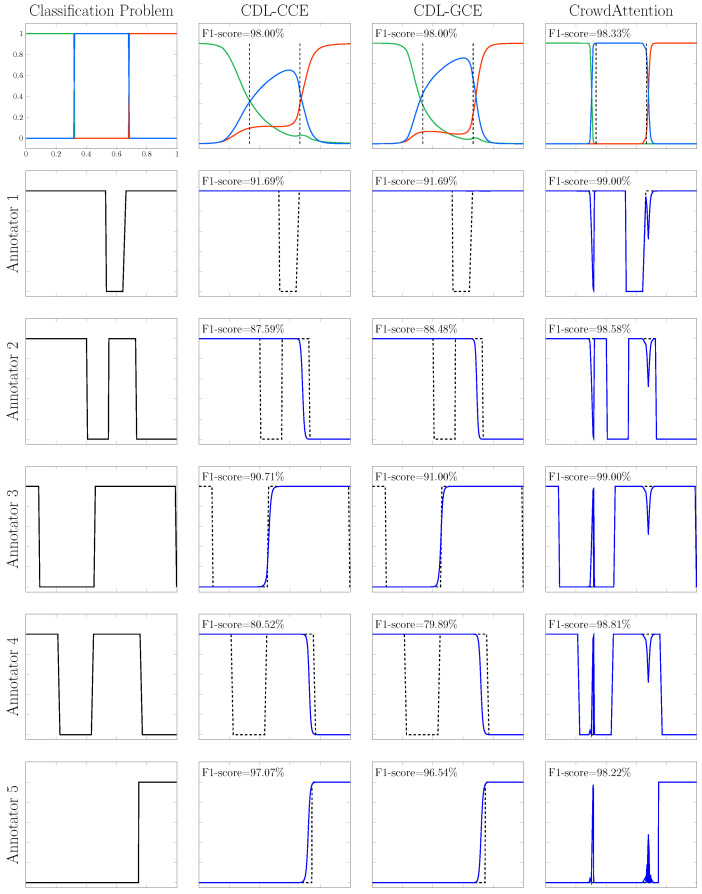
Results over fully-synthetic datasets. The first row, from left to right, presents the classification problem and the results for CDL-CCE, CDL-GCE, and CrowdAttention. In the first row, colors represent the predicted class probabilities for each class. Rows 2–6 show the reliability results (in terms of the $F_{1}$-score) for fully-synthetic datasets; we only consider those methods that measure the annotators’ reliability as a function of the input features (CDL-CCE and CDL-GCE). The dashed line denotes the ground-truth reliability, while the blue line indicates the reliability estimated by each method.

The strong performance of our model highlights its ability to effectively capture annotator reliability through the attention mechanism, enabling accurate label estimation and robust final predictions. To empirically support this claim, Figure 2 illustrates the predicted class probabilities ${\hat{y}}_{*,k}=p\left(y_{*}=k|x_{*}\right)$ alongside the instance-dependent reliability scores $p_{n}^{\left(w\right)}$ inferred by the cross-attention module. These scores directly represent the trust assigned to each annotator and thus provide a transparent interpretation of how pseudo-labels are formed. We compare our model’s output against CDL-CCE and CDL-GCE, which are state-of-the-art methods for modeling annotator reliability. We remark that the results in Figure 2 correspond to the best-performing run from the 10 iterations.

As shown in Figure 2, *CrowdAttention* achieves superior classification performance compared to the baseline methods. Specifically, our model attains the highest overall $F_{1}$-score of 98.33%, outperforming CDL-CCE and CDL-GCE, which both reach 98.00%. A qualitative inspection further supports this result: the class probability estimates produced by *CrowdAttention* align more closely with the actual decision boundaries defined in the synthetic setup (see the first column of Figure 2), resulting in sharper and more accurate predictions. A key factor behind this improved performance lies in the model’s ability to infer annotator reliability as a function of the input. As illustrated in the last row of Figure 2, *CrowdAttention* successfully recovers a diverse set of reliability patterns, including sharp transitions (annotators 1 and 3), non-monotonic behaviors (annotators 2 and 4), and sparse reliability signals (annotator 5). In contrast, CDL-CCE and CDL-GCE produce overly smoothed estimates that fail to capture important local variations in reliability. To quantitatively assess this capability, we compute the average $F_{1}$-score between the estimated and true annotator reliability functions. *CrowdAttention* achieves a score of 98.72%, substantially outperforming CDL-CCE (89.51%) and CDL-GCE (89.52%), further confirming its effectiveness in modeling complex annotator behaviors.

On the other hand, the estimated annotator reliability functions produced by *CrowdAttention* exhibit sharp fluctuations near class transition regions, as seen in the last row of Figure 2. To explain this behavior, we recall that in our model, the actual label is estimated from crowdsourced data using a cross-attention mechanism, where reliability scores are computed via the dot product between the model’s predicted class distribution (query) and each annotator’s one-hot label (keys and values). Thus, such ground truth estimation heavily relies on a proper computation of the model’s predictions, which can be inaccurate in regions near decision boundaries, thereby causing errors in the attention weights assigned to each annotator [44].

### 5.2. Real-World Datasets Results

Up to this point, we have presented empirical evidence that our approach provides a suitable representation of annotator behavior, resulting in competitive classification performance compared to state-of-the-art models. Such an outcome is a consequence of modeling the annotators’ performance as a function of the input features, allowing the model to adapt to the heterogeneity inherent in real-world labelers [22]. However, the experimental setup considered so far is based on synthetic labels, which may introduce biases derived from the simulation procedure itself. To address this limitation and further validate the generalization capacities of our method, we now turn to fully real-world datasets. These datasets present a more challenging scenario in which both the input samples and the annotations originate from real clinical applications.

Table 3 reports the performances of all methods in terms of the $F_{1}$-score; for multiclass settings, we report the macro-averaged $F_{1}$-score. Firstly, regarding the Voice Dataset, we observe that for scales G and R, all the approaches considered achieve comparable performance. We attribute this outcome to the high-quality annotations provided by the labelers, as studied in [45]. Notably, for scale G, the theoretical lower bound (DNN-MV) slightly outperforms DFLC-MW, confirming the observation that annotators exhibit reliable behavior. In contrast, scale B presents a more challenging scenario. Here, our CrowdAttention obtains the highest performance, which is a remarkable outcome given the greater uncertainty introduced by annotators, as perceptual scale B is known to be difficult to assess, even for trained specialists.

Next, we turn to the results obtained on the Histology dataset, which poses an even more demanding classification task due to its highly imbalanced label distribution (Tumor: 37,260; Stroma: 27,668; Immune Infiltrate: 10,315). In terms of overall performance, *CrowdAttention* outperforms all competing methods and achieves an $F_{1}$-score of 75.06, coming closest to the theoretical upper bound provided by DNN-GOLD (75.57). Beyond the global result, a per-class analysis reveals further advantages of our approach. Specifically, *CrowdAttention* achieves the best $F_{1}$-score on the *Tumor* class, outperforming all other models, including CDL-CCE and CDL-GCE, which are its most direct competitors as they also model annotator reliability as a function of the input features. Our proposal also performs competitively on the minority classes (*Stroma* and *Infiltrate*). These results empirically demonstrate the ability of our method to handle multi-labelers even in the presence of unbalanced classes.

Finally, Table 4 presents the results for the LabelMe and Music real-world datasets. Again, CrowdAttention also demonstrates strong generalization across diverse modalities. On the LabelMe dataset, which contains natural images annotated through crowdsourcing, our framework achieved an $F_{1}$-score of 82.19, outperforming all competing baselines and approaching the upper bound set by DNN-GOLD (89.12). This result highlights the attention mechanism’s ability to weigh annotator reliability, even in subjective visual tasks, effectively. Note that the CDL-based baselines (CDL-CCE and CDL-GCE) were evaluated using the top 40 labelers with the most labeled instances owing to memory constraints. Similarly, for the Music Genre Classification dataset, CrowdAttention reached an $F_{1}$-score of 69.61, again surpassing classical aggregation methods (DNN-MV and DNN-DS) and crowd-layer approaches, while reducing the performance gap with DNN-GOLD (75.42). These findings emphasize that the proposed model not only adapts to complex biomedical tasks but also extends its robustness to other domains where annotator disagreement and subjectivity are typical. Nonetheless, the remaining gap with respect to the upper bound indicates that future refinements, such as modeling systematic biases or incorporating richer annotator priors, may further improve performance.

Finally, we conducted a non-parametric Friedman test to evaluate the statistical significance of the results. The null hypothesis assumes equivalent performance among all multi-labeler algorithms [46], excluding the upper bound DNN-GOLD. Using a significance level of α=0.01, we obtained a Chi-square value of 24.03 with a *p*-value of 0.0042. Since p<0.01, we reject the null hypothesis and conclude that there are statistically significant differences among the evaluated algorithms.

### 5.3. Varying the Number of the Annotators

**Figure 3:**
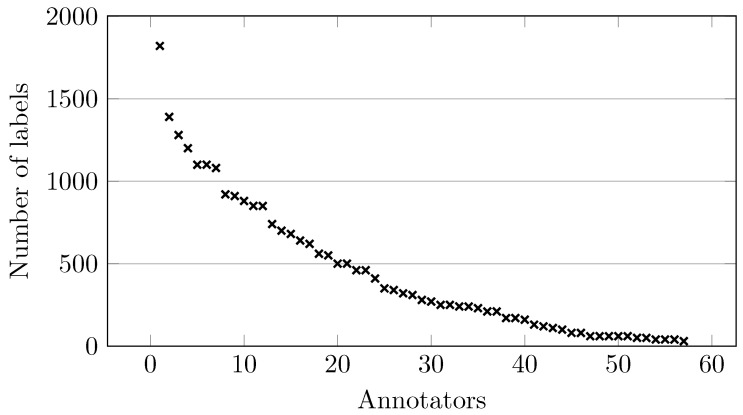
Number of labeled instances per annotator (sorted in descending order).

As a final experiment, we wish to evaluate the impact of the number of annotators on the performance of the multi-labeler classifiers. Specifically, we utilize the *LabelMe* dataset, which comprises 57 real annotators; we selected this dataset because it provides the largest number of labelers. The annotators were sorted in descending order according to the number of instances they labeled. Figure 3 shows the number of samples labeled by each annotator.

From Figure 3, we can observe a strong sparsity in the number of annotations per worker. Only a small group of annotators provided a large number of labels; in fact, less than 10 workers labeled more than 1000 samples (approximately 10% of the whole dataset). This distribution indicates that while a few annotators dominate the dataset in terms of volume, most provide only sparse information. Such an imbalance makes the learning problem more challenging, since the classifier must leverage a small fraction of highly active annotators while still exploiting the sparse signals from the long tail of less active ones.

**Figure 4:**
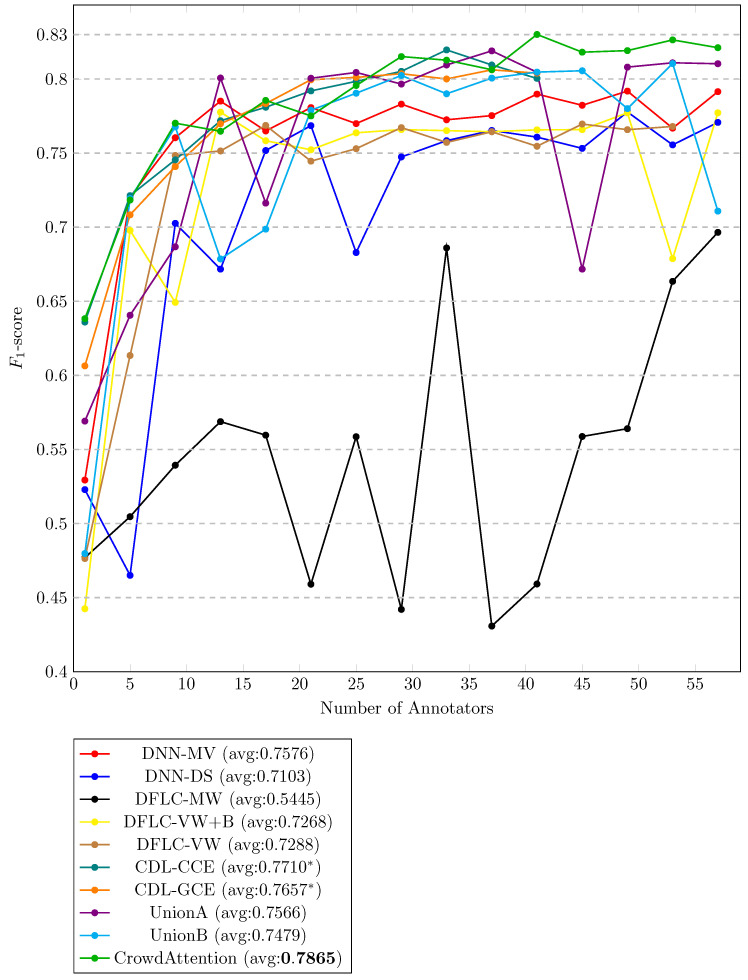
Generalization performance ($F_{1}\text{-}$score) as a function of the number of annotators. The number of annotators was increased in steps of four. Bold: the method with the highest average performance. We remark that for methods CDL-CCE and CDL-GCE (marked with an asterisk), the maximum number of annotators was 41 due to memory limitations.

To evaluate the effect of progressively increasing the number of annotators. Instead of incorporating them one by one, we adopted a cumulative scheme with a step size of four. In the first iteration, the system was trained and evaluated using only the labels from the most prolific annotator. In the second iteration, the first five annotators were included; in the third iteration, the first nine, and so forth, until all 57 annotators were aggregated. Figure 4 shows the classifiers’ performance in terms of F1 score as a function of the number of annotators. First, we notice that most approaches improve as more annotators are aggregated, with the most significant gains occurring in the initial increments (e.g., from 1 to 9 annotators). After this point, the performance tends to saturate, reflecting diminishing marginal benefits when adding additional annotators. Such an outcome is expected since the first annotators were those who contributed the most significant number of labels.

We also observe that methods based on label aggregation (DNN-MV and DNN-DS) quickly stabilize around a moderate level of performance. In fact, the simplest aggregation strategy achieves better results than the DFLC variants. On the other hand, we remark that the Union methods [5] consistently outperform the previous approaches; however, we highlight an instability in their progression. Namely, they present considerable drops in performance at certain points.

In contrast, the CDL-based approaches (CDL-CCE and CDL-GCE) exhibit more stable behavior across different numbers of annotators. Both methods maintain a relatively smooth progression without abrupt drops and consistently achieve competitive performance. We attribute such stability to the fact that these approaches are designed to model the annotators’ performance as a function of the input space, which makes them more robust to variations in the number of annotators and the level of label sparsity. However, a key limitation of CDL methods is that they allocate a separate network for each annotator, which makes them inherently non-scalable. In practice, this design imposes heavy memory requirements, to the point that it was only feasible to run experiments with up to 41 annotators.

Finally, *CrowdAttention* achieves the best overall results, surpassing all baselines while maintaining a stable progression. Similar to CDL approaches, it models annotator performance as a function of the input instances; however, in contrast to CDL, *CrowdAttention* relies on a dot-product attention mechanism. This design enables the model to scale efficiently with the number of annotators, avoiding the memory limitations of CDL methods while continuing to benefit from the contributions of additional annotators. As a result, *CrowdAttention* achieves both higher and more consistent performance across the entire range.

### 5.4. Limitations

Although our CrowdAttention approach demonstrates competitive performance across synthetic and real-world datasets, several limitations remain. First, in the synthetic experiments, we observed sharp fluctuations in the estimated annotator reliability functions near class transition regions (see Figure 2). This behavior arises because the cross-attention module relies on the model’s predicted class distribution to compute reliability weights, making the approach sensitive to misclassifications close to decision boundaries. Second, while CrowdAttention avoids the heavy parameterization of annotator-specific confusion matrices, it does not explicitly capture structured error patterns such as systematic biases or class-dependent confusions, which were evident in challenging cases of the Voice dataset—particularly for the “Breathiness” (B) dimension (see Table 3), where annotators displayed high disagreement and our model’s advantage over baselines was more modest. Third, in the histopathology dataset, although CrowdAttention achieved the highest overall F1-score among competing methods (75.06 vs. 75.57 for the upper bound DNN-GOLD), its performance on minority classes such as Stroma and Immune Infiltrates remained affected by the strong class imbalance, highlighting reduced robustness under skewed distributions (see Table 3). Finally, while our attention-based aggregation is more scalable than annotator-specific models, effective training still requires careful tuning of the classification backbone to avoid overfitting to noisy supervision, especially when the number of annotators is limited.

## 6. Conclusions

In this work, we present *CrowdAttention*, a novel framework for learning from crowdsourced annotations. Unlike prior approaches that rely on explicitly modeling annotator confusion matrices or estimating per-annotator parameters, *CrowdAttention* introduces a compact and scalable architecture based on attention mechanisms, enabling the estimation of annotator reliability as an instance-dependent quantity. Our method combines two interconnected modules: a classification network that predicts class distributions based on the input features, and a crowd network that infers soft pseudo-labels from noisy annotations via a cross-attention mechanism. This design enables the model to adaptively weight annotator inputs based on their agreement with the model’s prediction, resulting in a flexible and interpretable estimate of annotators reliability. This makes it especially attractive for large-scale real-world applications such as medical diagnosis, where high-quality labels are scarce or expensive [2]. Experimental results with synthetic and fully real datasets confirm that our CrowdAttention accurately captures annotator expertise and achieves high classification performance. Moreover, the proposed architecture scales linearly with both the number of annotators and classes, ensuring computational efficiency and making it well suited for large-scale crowdsourcing scenarios.

As future work, we plan to follow the ideas presented in [2,16] to extend our work by estimating a per-annotator matrix of confusion, which will enhance the representation of annotators’ behavior. Furthermore, we plan to adapt our approach to address scenarios involving Multiple Instance Learning. Here, rather than multiple annotators, we have to deal with multiple labels from the instances inside a bag. This approach could significantly improve the diagnosis and treatment of behavioral disorders [47].

## Figures and Tables

**Table 1:** Comparisons between CrowdAttention and representative multi-annotator learning methods. “End-to-End” indicates whether the method trains the classifier and the annotator model jointly in a single optimization step. “Input-Aware” specifies if the method leverages input features when estimating annotator reliability, rather than assuming it is fixed. “Parametric-Efficiency” denotes whether the method avoids assigning a large set of parameters to each annotator and instead uses a more compact and scalable parameterization.

Method	End-to-End	Input-Aware	Parametric-Efficiency
Majority Voting (MV) [27]	✗	✗	✓
Dawid–Skene (DS) [12]	✗	✗	✗
GLAD [32]	✗	✗	✗
CrowdLayer [15]	✓	✗	✗
Knowledge Transfer [18]	✓	✓	✗
Chained-NN [20,22]	✓	✓	✗
UnionNet [5]	✓	✗	✓
CrowdAttention (ours)	✓	✓	✓

**Table 2:** Comparison of baseline algorithms. DNN: deep neural network. MV: majority voting. DFLC: deep learning from crowds. CDL: chained deep learning.

Algorithm	Description
DNN-GOLD	A DNN trained using the real labels (upper bound performance).
DNN-MV	A DNN trained using majority voting over annotators as the ground truth.
DNN-DS	A DNN trained on labels aggregated by the classical EM-based method proposed in [12].
DLFC [15]	A Crowd Layer for deep learning assuming homogeneous workers. We consider three variants: MW (Majority Weighted), VW (Voting Weighted), and VW+B (Voting Weighted with Bias).
CDL-CCE [20]	A regularized chained deep neural network for multiple annotators, based on cross-entropy loss (CCE). Assumes labelers with heterogeneous performance.
CDL-GCE [22]	Improvement of CDL-CCE that incorporates a robust loss function based on Generalized Cross Entropy (GCE) (q=0.1).
UnionNet [5]	A Crowd Layer for deep learning, which integrates the information from multiple annotators through concatenation. We evaluate two variants, referred to as UnionA and UnionB.

**Table 3:** Results on Voice and Histology datasets with real-world annotators, reported in terms of the $F_{1}$-score. Bold: the method with the highest performance excluding the upper bound classifier DNN-GOLD.

	Voice Dataset			Histology			
	G	R	B	Global Result	Per-Class		
					Tumor	Stroma	Infiltrate
DNN-GOLD	85.64±5.54	85.64±5.54	85.64±5.54	75.57	84.66	71.12	70.93
DNN-MV	79.09±6.62	74.05±3.60	42.41±7.58	72.69	85.80	68.78	63.49
DNN-DS	74.01±6.00	74.41±4.76	50.97±6.85	73.58	86.03	70.61	64.10
DLFC-MW	78.81±7.65	80.03±6.41	58.66±7.41	66.81	79.13	59.13	62.18
DLFC-VW	82.17±7.36	81.44±4.32	60.37±8.24	68.28	79.23	68.78	63.49
DLFC-VW+B	81.32±4.34	80.33±4.69	62.39±7.15	68.41	79.00	60.07	66.16
CDL-CCE	82.22±7.58	83.11±4.96	71.30±7.58	70.48	80.25	63.94	67.25
CDL-GCE	82.63±6.04	82.74±5.75	70.64±7.58	69.96	80.48	63.39	66.00
UnionA	80.56±8.89	79.03±5.54	55.79±8.09	71.96	82.81	68.83	64.24
UnionB	69.53±8.75	72.55±9.18	38.36±9.80	72.02	83.74	68.51	63.79
CrowdAttention	82.09±4.80	82.55±6.06	74.58±7.07	75.06	86.49	70.73	67.97

**Table 4:** Results on LabelMe and Music datasets with real-world annotators, reported in terms of the $F_{1}$-score. Bold: the method with the highest performance, excluding the upper bound classifier DNN-GOLD. Note that for CDL-CCE and CDL-GCE (marked with an asterisk), we restricted the analysis to the top 40 labelers with the most labeled instances because of memory limitations.

	LabelMe	Music
DNN-GOLD	89.12	75.42
DNN-MV	77.78	65.77
DNN-DS	77.08	13.00
DLFC-MW	55.55	65.06
DLFC-VW	76.84	65.81
DLFC-VW+B	78.08	62.82
CDL-CCE	81.26 *	64.39
CDL-GCE	78.99 *	63.21
UnionA	80.99	67.91
UnionB	78.48	56.61
CrowdAttention	82.19	69.61

## Data Availability

Publicly available datasets were analyzed in this study. These datasets can be found in the following repositories: the LabelMe and Music datasets are available at https://fprodrigues.com (accessed on 8 October 2025), and the Histology dataset is available at https://github.com/wizmik12/DGPCR?tab=readme-ov-file (accessed on 8 October 2025). The Voice dataset is not publicly available due to privacy restrictions.
